# Validity and reliability of the Norwegian version of the Musculoskeletal Health Questionnaire in people on sick leave

**DOI:** 10.1186/s12955-021-01827-4

**Published:** 2021-08-03

**Authors:** Alexander Tingulstad, Maurits W. Van Tulder, Tarjei Rysstad, Anne Therese Tveter, Jonathan C. Hill, Margreth Grotle

**Affiliations:** 1 Department of Physiotherapy, Faculty of Health Sciences, Oslo Metropolitan University, Pilestredet 44, 0167 Oslo, Norway; 2 Department Human Movement Sciences, Faculty of Behavioural and Movement Sciences, Amsterdam Movement Sciences Research Institute, Vrije Universiteit, Van der Boechorststraat 7-9, 1081 BT Amsterdam, the Netherlands; 3 National Advisory Unit On Rehabilitation in Rheumatology, Diakonhjemmet Hospital, P.B. 23 Vinderen, 0319 Oslo, Norway; 4 School of Medicine, Primary Care Centre Versus Arthritis, Keele University, Staffordshire, ST5 5BG UK; 5 Oslo University Hospital, Research and Communication Unit for Musculoskeletal Health, P.B. 4950 Nydalen, 0424 Oslo, Norway

## Abstract

**Background:**

The Musculoskeletal Health Questionnaire (MSK-HQ) is a recently developed generic questionnaire that consists of 14 items assessing health status in people with musculoskeletal disorders. The objective was to translate and cross-culturally adapt the MSK-HQ into Norwegian and to examine its construct validity and reliability in people on sick leave with musculoskeletal disorders.

**Methods:**

A prospective cohort study was carried out in Norway on people between 18 and 67 years of age and sick leave due to a musculoskeletal disorder. The participants were recruited through the Norwegian Labour and Welfare Administration during November 2018–January 2019 and responded to the MSK-HQ at inclusion and after four weeks. Internal consistency was assessed by Cronbach’s alpha, and structural validity with a factor analysis. Construct validity was assessed by eight “a priori” defined hypotheses regarding correlations between the MSK-HQ and other reference scales. Correlations were analyzed by Spearman’s- or Pearson’s correlation coefficient and interpreted as high with values ≥ 0.50, moderate between 0.30–0.49, and low < 0.29. Reliability was tested with test–retest, standard error of measurement (SEM) and smallest detectable change (SDC).

**Results:**

A total of 549 patients, mean age (SD) 48.6 (10.7), 309 women (56.3%), were included. The mean (SD) MSK-HQ sum scores (min–max 3–56) were 27.7 (8.2). Internal consistency was 0.86 and a three-factor structure was determined by factor analysis. Construct validity was supported by the confirmation of all hypotheses; high correlation with HRQOL, psychosocial risk profile, and self-perceived health; moderate correlation with physical activity, self-perceived work ability, and work presenteeism; and low correlation with the number of sick days. The test–retest reliability was good with an intraclass correlation coefficient of 0.83 (95% CI, 0.74–0.89), SEM was 2.3 and SDC 6.5.

**Conclusions:**

The Norwegian version of the MSK-HQ demonstrated high internal consistency, a three-factor structure**,** good construct validity and good test–retest reliability when used among people on sick leave due to musculoskeletal disorders.

## Introduction

Musculoskeletal (MSK) disorders cause major health challenge and burden for individuals, health systems and social care worldwide [1, 2]. General MSK disorders such as osteoarthritis, inflammatory conditions, back, neck, shoulder and knee pain have been the single greatest cause of years lived with disability for many years [1].

Patients with MSK pain in different body regions tend to share the same prognostic factors, supporting the use of generic patient-reported outcome measures (PROMs) [3, 4]. The lack of generic questionnaires for people with MSK disorders argues for development and validation of new tools for assessment of this wide group of disorders. Due to the similar characteristics and prognostic factors for MSK disorders one questionnaire might be sufficient to assess necessary contents. The Musculoskeletal Health Questionnaire (MSK-HQ) is a recently developed PROM that assesses health status in people with MSK disorders [5]. The questionnaire is one of few tools made specifically for assessment of MSK disorders, and given its generic form, it is appropriate for assessing and comparing numerous MSK disorders [6]. MSK-HQ is available in English and besides the original study, two studies have performed validation of the English questionnaire [7, 8]. The development of the MSK-HQ included a scoping exercise to identify domains important for MSK health and a qualitative evaluation of face and content validity [5]. Further on, assessment of construct validity, test–retest reliability and data quality have been conducted with acceptable results [5, 7, 9]. To expand the use of MSK-HQ to Norway, a translation process and assessment of its psychometric properties are essential. Therefore, the aim of this study is to translate and cross-culturally adapt the MSK-HQ into Norwegian and to examine its validity and reliability in people on sick leave due to musculoskeletal disorders.

## Methods

### Translation and cross-cultural adaption

The MSK-HQ is a generic questionnaire that consists of 14 questions concerning MSK health during the last two weeks, including domains as pain, fatigue, physical function, sleep, self-efficacy, and psychological well-being [5]. The questions are answered using a 5-point ordinal scale, from “not at all” (4) to “extremely” (0). Questions 12 and 13, concerning self-efficacy and health literacy, have their response in reversed order were “not at all” is scored zero. A 15th question, which is not part of the MSK-HQ total score, captures physical activity levels and is scored from 0 to 7. A sum of the first fourteen questions is used to calculate the MSK-HQ total score, and gives a result between 0 and 56, with higher scores reflecting better MSK health status.

The original English version of the MSK-HQ was translated into Norwegian after gaining approval from the original authors of the MSK-HQ [5]. The translation and cross-cultural adaption followed the guidelines proposed by Beaton et al., consisting of 6 stages [10]. In the first stage, two Norwegian translators fluent in English translated the items into Norwegian independently. The second stage consisted of a synthesis of the two translations. In the third stage, a back-translation was performed individually by two native English bilingual speakers. At the fourth stage, an expert committee consisting of four translators, four of the authors of this article and an additional two researchers from our research project reviewed the previous translations and reached a consensus on discrepancies before a prefinal version was produced. In the fifth stage, the prefinal version was completed by 25 people with MSK disorders who commented on all aspects of the questionnaire. On the sixth and last step, the final version of the questionnaire was completed and submitted to the developers of the original questionnaire.

### Design and study population

The study was a prospective cohort study of people on sick leave due to MSK disorders [11]. The participants were recruited through the Norwegian Labour and Welfare Administrations (NAV) webpages during November 2018–January 2019. Eligible participants were invited to read project information and to consent electronically to participate. There were not any registration of the number of people that rejected the opportunity to participate. Eligible participants were people between 18 and 67 years of age and on sick leave for at least four weeks due to an MSK disorder. People on sick leave for other disorders or diseases or people not able to understand and write Norwegian or English were excluded. All participants agreed to consent before being admitted to the study. The participants answered the questionnaire electronically at baseline, and were asked to respond a second time after four weeks. The MSK-HQ was part of a comprehensive questionnaire used in the study. This study was carried out in two phases. First a translation and cross-cultural adaption of the original MSK-HQ into Norwegian. Second, the Norwegian version of the MSK-HQ was tested for its psychometric properties.

### Measurements

In addition to the Norwegian version of the MSK-HQ (including item 15 on physical activity), the baseline questionnaire consisted of sociodemographic data, medical history, MSK diagnosis (ICPC-2), and different PROMs, and is presented in the published protocol [11]. Several reference scales and single items were used to evaluate construct validity: first, the EuroQol 5 Dimensions (EQ-5D-5L), which covers five dimensions within health-related quality of life (HRQOL): mobility, self-care, daily activities, pain/discomfort and anxiety/depression [12]. Second, the Örebro Musculoskeletal Pain Screening Questionnaire short form (ÖMPSQ-SF), which is a widely used screening questionnaire used for early identification of yellow flags and patients at risk of developing work disability due to pain [13]. Third, the Keele Subgroups for Targeted Treatment (the Keele STarT MSK) tool, which is a questionnaire developed to assess risk of poor outcome and enable risk stratification for people with MSK disorders [14, 15]. The single items are the EQ-5D Visual Analogue Scale (0–100 VAS), from the EQ-5D-5L assessing self-perceived health [12], presenteeism assessed with item 9 from the Institute for Medical Technology Assessment Productivity Cost Questionnaire (iPCQ) [16], and a self-perceived work ability question ranging from 0–10 [17]. Additionally, the participants’ number of sick days for the last 12 months were summarized using registry data from the NAV. The number of sick days was measured as calendar days and adjusted for percentage of sick leave.

For test–retest purposes, a follow-up questionnaire was collected four weeks after baseline that contained the same questions as in the baseline questionnaire. In addition, it included a seven-point global rating of change scale, ranging symptoms from “very much worse” to “much better”. To ensure that change in symptoms would not influence the results, only participants rating “no change” in symptoms were included in the test–retest subgroup and the assessment of test–retest reliability.

### Statistical analysis

IBM SPSS Statistics for Windows, version 26.0 (IBM Corp, Armonk, NY) was used for all data analyses. Descriptive analyses included means (SD) and frequencies (%). The sample size was based on the quality criteria suggested by Terwee et al., with at least 50 participants needed for assessing interpretability with floor or ceiling effects, and minimum 100 people for assessing internal consistency and construct validity, and conducting factor analysis [18]. The distribution of normality was assessed with the Kolmogorov–Smirnov test and by visual inspection of the distribution plot. Construct validity was assessed by formulating and testing of eight “a priori” defined hypotheses regarding the correlation between MSK-HQ and other constructs [19]. Test–retest reliability was determined by calculating the single measures intraclass correlation coefficient (ICC) with a two-way random effects model, absolute agreement (2,1). An ICC value above 0.7 was considered acceptable [18].

Factor analysis by a principal component analysis was performed on the results to determine structural validity. Retained factors had an eigenvalue > 1, and independent factors were obtained by the use of oblique rotation, direct oblimin.

The internal consistency reflects the interrelatedness among the items of the questionnaire, and the interrelatedness in the MSK-HQ was assessed with inspection of inter-item correlations and with Cronbach’s Alpha [19]. The value of Cronbach’s Alpha ranges from 0 to 1 and is considered acceptable when between 0.7 and 0.95 [18]. High values > 0.95 reflects high correlations between the items in the questionnaire and may indicate redundancy of one or more items [18].

The distribution of normality determined if parametric (Pearson’s correlation coefficient) or nonparametric (Spearman’s rank correlation coefficients) were used to assess the correlation between the constructs. The correlation coefficients were interpreted as being high when > 0.5, moderate when between 0.30–0.49, and low when < 0.29 [20]. The hypotheses used to assess construct validity were established based on the construct of the measures and former correlations of similar constructs. The MSK-HQ was hypothesized to have high correlation with the EQ-5D-5L, the ÖMPSQ-SF and self-rated health. A moderate to high correlation was expected with the Keele STarT MSK, productivity loss, self-perceived work ability, and physical activity. A moderate to low correlation was hypothesized with sick leave 12 months before baseline. Construct validity was considered acceptable if 75% of the “a priori” hypotheses were confirmed [18].

Measurement error was assessed with standard error of measurement (SEM) and smallest detectable change (SDC). The formula used for SEM was SEM = SD_difference_ ÷ √2 and for SDC was SDC = SEM × 1.96 × √2 [21]. The agreement between the test and retest scores was assessed with a Bland–Altman plot and the limits of agreement (95%) were calculated by the formula [mean difference ± 1.96 × SD_difference_] [22].

To determine interpretability, floor and ceiling effects were analysed, and considered present if more than 15% of the participants scored the lowest or highest score, respectively [18, 23]. The number of participants with the lowest and highest score for each of the items was also reported.

## Results

### Translation and cross-cultural adaption

During the forward and backward translations of the MSK-HQ, the expert committee found that the Norwegian MSK-HQ was generally clear and understandable except for a few minor vocabulary adaptions to words and expressions in the items 1, 5, 6, 11 and 13. The preliminary Norwegian translation of the words “severe”, “unable”, “interfered”, and “low in your mood” (item 1, 5, 6, 11) were altered by the committee, as well as the heading of item 13 (confidence in being able to manage your symptoms). The pilot testing on 25 people with MSK disorders did not result in any changes in the wordings in the MSK-HQ. The Norwegian MSK-HQ is presented in Appendix 1.

### Participants and data quality

A total of 549 people completed the questionnaires online. Table 1 presents sociodemographic data and clinical variables for the whole sample and the test–retest subgroup at baseline. The MSK-HQ items were all mandatory and participants were unable to go on without complete answers. Hence, there were no missing values in the questionnaire. The mean score of the MSK-HQ was 27.7 (8.2). The lowest score obtained was 3 points, while one person reached the highest score possible (56), indicating no floor or ceiling effects of the full questionnaire, presented in Table 2. There were three single items (3, 4 and 9) were > 15% of the participants answered the lowest or highest possible value (Table 2).

**Table 1:** Baseline characteristics of the participants

Mean (SD) or N (%)	Whole sample (n = 549)	Test–retest subgroup (n = 101)
Age (yrs.)	48.6 (10.7)	48.8 (10.4)
Gender (% Women)	309 (56.3)	57 (56.4)
Civil status (%)		
Married/cohabiting	426 (77.6)	76 (75.2)
Single/divorced	75 (13.7)	25 (24.8)
Education (> 12 years) (%)	220 (40.1)	39 (38.6)
L-diagnosis (ICPC-2) (%)		
Lower limb (L13-17)	47 (8.6)	10 (10.0)
Upper limb (L08-12, L92-93)	121 (22.0)	16 (16.0)
Neck (L01, L83)	36 (6.7)	3 (3.0)
Low back (L02-03, L70, L84-86)	107 (19.5)	21 (20.9)
Joint disorders (L88-91)	54 (9.8)	14 (13.9)
Injuries and trauma (L72-81, L96)	51 (9.3)	7 (7.0)
Other MSK diagnoses (L05, L07, L18-20, L26-29, L71, L82, L87, L94-95, L97-99)	133 (24.2)	30 (29.7)
Self-perceived work ability (0–10), median (range)	3 (0–10)	3 (0–9)
Self-rated health status/ EQ-VAS (0–100), mean (SD)	52.0 (21.1)	45.8 (21.0)
Physical activity (MSK-HQ q15), median (range)	2 (0–7)	1 (0–7)
Sick days the last year, median (range)	37.8 (2.3–239.2)	59.6 (13.1–237.1)
MSK-HQ (0–56), mean (SD)	27.7 (8.2)	24.9 (8.3)
Keele STarT MSK (0–12), mean (SD)	7.0 (2.4)	7.9 (2.0)
ÖMPSQ-SF (0–100), mean (SD)	55.4 (15.6)	61.8 (15.1)
iPCQ Q9 presenteeism (0–10), median (range)	5 (0–10)	5 (2–10)
EQ-5D-5L (− 0.59–1), median (range)	0.56 (− .35–1)	0.42 (− .35–.80)

ICPC-2 = International Classification of Primary Care, 2^nd^ edition; MSK-HQ = Musculoskeletal Health Questionnaire; ÖMPSQ-SF = Ôrebro Musculoskeletal Pain Screening Questionnaire short form; iPCQ = iMTA Productivity Cost Questionnaire

**Table 2:** Descriptive statistics, floor and ceiling effects of the MSK-HQ items

Item	Mean (SD)	Item-total correlation	Lowest score (%)	Highest score (%)
1	1.39 (0.7)	.59	8.2	0.5
2	1.83 (1.0)	.57	7.3	4.6
3	2.44 (1.2)	.46	1.6	26.6
4	2.84 (1.0)	.48	0.2	32.2
5	1.87 (1.2)	.53	10.4	10.9
6	1.40 (0.8)	.57	9.8	0.7
7	1.77 (1.0)	.64	8.6	5.1
8	2.26 (1.0)	.53	1.6	14.2
9	1.57 (1.2)	.54	22.4	6.7
10	1.82 (1.1)	.57	8.4	8.0
11	2.32 (1.1)	.52	3.4	14.2
12	2.60 (1.0)	.20	3.1	13.7
13	2.21 (0.9)	.36	4.2	3.5
14	1.39 (0.7)	.68	7.1	0.2

MSK-HQ = Musculoskeletal Health Questionnaire

**Table 3:** Results of the explanatory factor structure by principal component analysis with loadings (n = 549)

Item		Factor 1	Factor 2	Factor 3
1	Pain/stiffness during the day	0.45		
2	Pain/stiffness during the night			− 0.86
3	Walking	0.85		
4	Washing/dressing			− 0.39
5	Physical activity levels	0.84		
6	Work/daily routine	0.65		
7	Social activities and hobbies	0.59		
8	Needing help	0.41		
9	Sleep			− 0.89
10	Fatigue or low energy			− 0.58
11	Emotional well-being		0.42	
12	Understanding of your condition and any current treatment		0.88	
13	Confidence in being able to manage symptoms		0.84	
14	Overall impact	0.48		

Extraction Method: principal component analysis; oblique rotation with Kaiser normalization. Values below 0.3 are suppressed. The model explained 60% of the total variance; factor 1 explained 38%, factor 2 = 12% and factor 3 = 10%

**Table 4:** Construct validity: “a priori” formulated hypothesis

Hypothesis	Correlation value	N	Hypothesis confirmed?
A high score on the MSK-HQ (good MSK health) is expected to have high correlation with high health-related quality-of-life assessed by EQ-5D-5L	.764	541	Yes
A low score on the MSK-HQ (poor MSK health) is expected to have high/moderate negative correlation with a high score on the Örebro Musculoskeletal Pain Questionnaire short form.	− .659	549	Yes
A low score on the MSK-HQ (poor MSK health) is high/moderate negative associated with high score on the Keele STarT MSK	− .689	549	Yes
A low score on the MSK-HQ (poor MSK health) is high/moderate associated with higher productivity losses.	.336	237	Yes
A low score on the MSK-HQ (poor MSK health) is high/moderate associated with low self-perceived work ability.	.412	548	Yes
A low score on the MSK-HQ (poor MSK health) is high associated with low self-rated health (EQ-VAS)	.592	542	Yes
A low score on the MSK-HQ, indicating poor MSK health, is high/moderate associated with few days with physical activity.	.378	535	Yes
A low score on the MSK-HQ (poor MSK health) is low/moderate associated with longer sick leave.	.001	549	Yes

MSK-HQ = Musculoskeletal Health Questionnaire; MSK = Musculoskeletal.

### Structural validity

Before the factor analysis was performed, the suitability of data was assessed. The correlation matrix showed presence of many coefficients above 0.3. The Kaiser–Meyer–Olkin value was above the recommended value of 0.6 (0.83) and the Bartlett’s test of sphericity demonstrated a significant value. The principal component analysis revealed that 3 factors exceeded eigenvalues of 1 (5.3, 1.6 and 1.3) explaining a total of 60% (38%, 12% and 10%) of the variance (Table 3). Inspection of the scree plot showed a clear break after the third factor. Item 1, 2, 3, 5–8 and 14 loaded most strongly on factor 1, item 11–13 loaded on factor 2, and item 2, 4, 9 and 10 loaded factor 3. The inter-total correlation of the MSK-HQ revealed that items 12 and 13 were less related to the other items, reflecting a different construct.

### Internal consistency

The internal consistency was considered good after inspection of inter-item correlations and a Cronbach’s alpha value of 0.86.

### Construct validity

Construct validity was assessed by testing eight “a priori” formulated hypotheses as presented in the left column of Table 4. Visual inspection of distribution plots and assessing Kolmogorov–Smirnov tests determined the normality distribution of the variables. The MSK-HQ, ÖMPSQ-SF, Keele STarT MSK, and self-perceived health were normally distributed, while physical activity and number of sick days were left skewed, EQ-5D-5L and self-perceived work ability were skewed right, and presenteeism was uniformly distributed. The correlation analysis (Table 4) demonstrated a high correlation between the MSK-HQ and EQ-5D-5L, ÖMPSQ-SF, Keele STarT MSK and self-rated health, a moderate correlation with presenteeism, self-perceived work ability and physical activity, and a low correlation with the number of sick days.

### Reliability

**Fig. 1:**
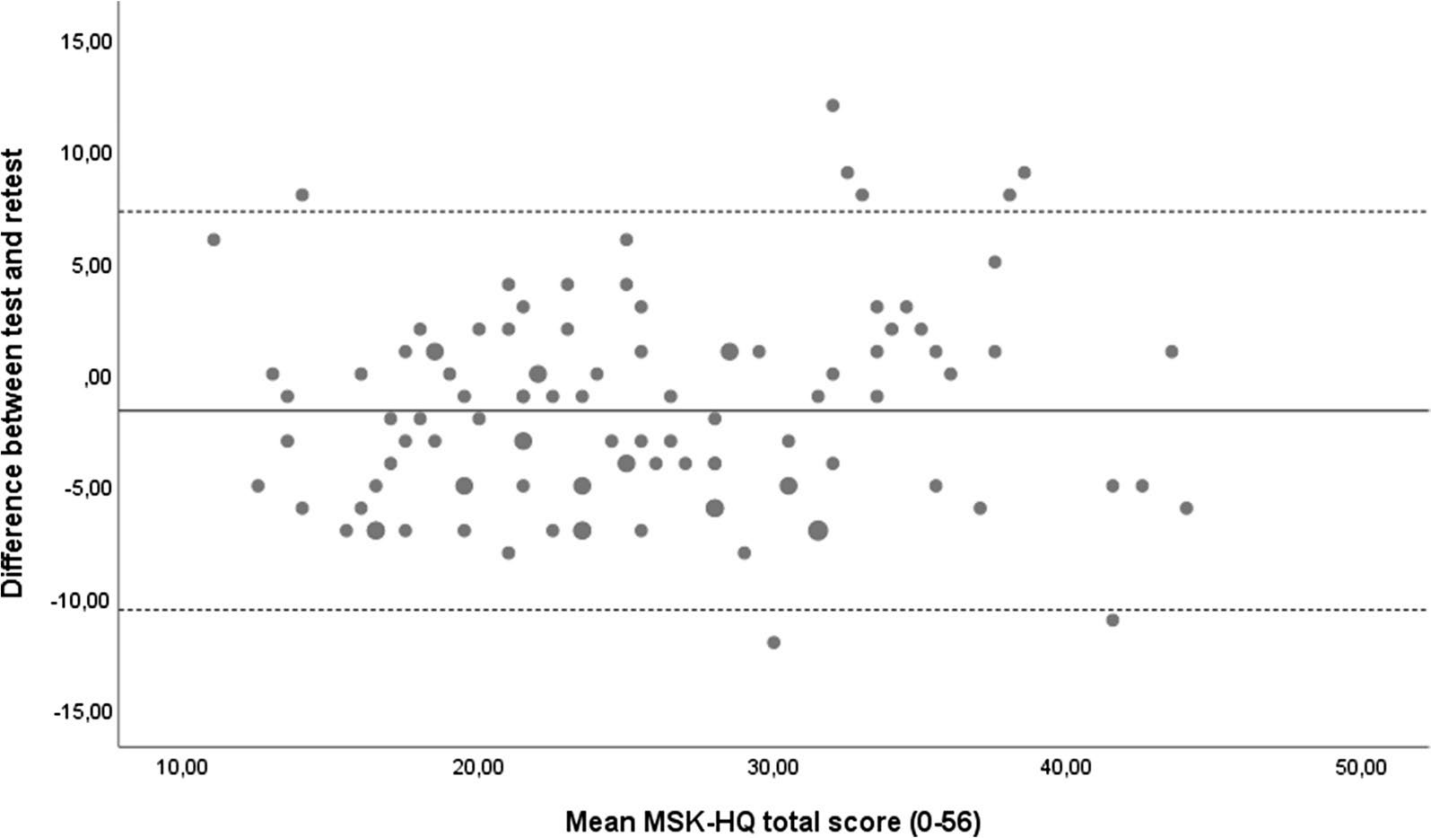
Bland–Altman plot of mean difference and the Limits of agreement. Enlarged circles represent two identical values

The mean (SD) time between test and retest was 31 (5.4) days, and the questionnaire was answered by 330 participants. There were 101 (31%) participants that scored “no change” in the muscle and joint symptoms for the last four weeks, while 47 (14%) participants reported worse symptoms and 182 (55%) participants reported improvement of symptoms. The total score of the MSK-HQ for the test–retest subgroup (N = 101) was mean (SD) 24.9 (8.3) at baseline and 26.5 (7.9) at retest. The ICC_2.1_ (95% CI) between test and retest was 0.83 (0.74–0.89). The calculation of measurement error resulted in an SEM of 2.33 and an SDC at 6.46. The mean difference between the test and retest was − 1.6 points, with limits of agreement of 7.26 and − 10.54 points (Fig. 1).

## Discussion

In this study, the Norwegian version of the MSK-HQ was translated and showed to be a valid and reliable instrument to measure MSK health in people on sick leave due to an MSK disorder. The translation and cross-cultural adaption were successfully accomplished according to international guidelines [10], and the psychometric properties in terms of structural and construct validity, internal consistency and reliability were found to be good. Our results indicate that the MSK-HQ can be used in both clinical settings and research with the purpose of assessing people with MSK disorders.

In this study, the total score showed no floor and ceiling effects, which is consistent with former studies assessing the MSK-HQ [5, 7, 9]. Although, when assessing floor and ceiling effects at single items in this population, item 3 (gait) and item 4 (washing/dressing) demonstrated floor effects and item 9 (sleep) showed a ceiling effect.

The total score of the MSK-HQ was quite similar between the Norwegian and the British study populations. The Norwegian population with different MSK disorders achieved a mean total score of 27.7, while the British populations scored a mean total score of 28.6 [5] and 26.6 [7]. The Danish population with MSK disorders, similar to our study, reached a higher mean score in both MSK-HQ (32.3) and EQ-5D-5L (0.69) [9]. One difference that might explain some of the differences is that only 7% of the Danish population was on sick leave and were possibly less affected of their MSK disorders [9]. Even though there are some differences, the scores are within the measurement error of the MSK-HQ.

The factor structure was investigated and revealed a clear three-factor structure explaining 60% of the total variance. The general items formed the first factor, health literacy, self-efficacy and emotional well-being composed the second factor, while the third factor consisted of fatigue and problems during the night. The three factors determined in these results might reflect how the questionnaire divides within three main domains or three subscales. Although, our results differs from a principal component analysis performed in a previous study, where a one factor structure explained 63% of the variance, and a latent minor variable explained 10% of the item variance [7]. The minor variable of 10% consisted of item 12 and 13 (health literacy and self-efficacy), which reflects similarities with the second factor of our study. Item 12 and 13 also achieved the lowest inter-total correlation value among the questionnaire, which is similar to previous research [7].

The Norwegian MSK-HQ demonstrated high internal consistency, although a somewhat lower value than previous studies [5, 7, 9]. The items of the scale seemed to have high interrelatedness despite the different domains.

Based on a priori hypotheses, the Norwegian MSK-HQ demonstrated good construct validity. Previous studies have investigated the correlation between the MSK-HQ and HRQOL, and similar to our study the analyses showed a high correlation with EQ-5D-5L ranging from 0.78 to 0.81 [5, 7]. The high correlation might reflect that the two questionnaires overlap in what they measure, hence the questionnaires can be redundant if used at the same patient or population. While the EQ-5D-5L with its five items is relatively fast to answer, the MSK-HQ contributes insight to many useful domains, especially for clinicians. The 15 items give clinicians the possibility to target the intervention or information towards certain domains within the MSK-HQ. The other correlation values presented in Table 4 are also somewhat as expected, considering the relatedness of the constructs. High and similar correlations with the screening questionnaires ÖMPSQ-SF and the Keele STarT MSK reflect that they are assessing many of the same aspects and items, such as self-perceived function, pain, and distress. The last hypothesis with a high correlation was self-rated health, which also has close relations to several of the items in the questionnaires. Also, the MSK-HQ showed moderate correlations to the different aspects of work, productivity and self-perceived work ability, whereas the lack of correlation with the number of sick days the last year is noteworthy. The broad range of domains in the MSK-HQ seems to have some overlap with the assessed questionnaires, although not redundant if used in a clinical setting or research. Due to the low correlation with former sick leave the questionnaire should not be used for predicting absenteeism. Due to few studies conducted concerning correlations between the MSK-HQ and other constructs, the eight hypotheses were rather broad, which could have influenced our chances of successfully confirming them. As the construct of MSK-HQ is investigated, further research should have the ability to specify and narrow down their hypotheses when assessing construct validity.

Assessment of the reliability resulted in a good test–retest in line with other studies conducted, which also demonstrated good test–retest reliability with ICC values of 0.73–0.86 [5, 7, 9]. Even though the test–retest subgroup reported “no change” in symptoms the last four weeks, a few outliers with are seen in Fig. 1. Particularly, more outliers and variation is seen in the upper end of the score. The measurement error of the MSK-HQ achieved good results with a low SDC of 6.46 points of the full range of the scale from 0–56. The implication of a low measurement error is that the questionnaire may be an appropriate outcome measurement to evaluate change during treatment or in a clinical setting. Although, one should keep in mind the variation seen in Fig. 1, and that the MSK-HQ demonstrated a somewhat higher estimate of measurement error in other populations [5, 7, 9], and further assessments are necessary to determine accurate values.

Our study conducted in a population on sick leave due to MSK disorders provides a good addition to previous research, which has been conducted on inflammatory arthritis, osteoarthritis and MSK disorders in primary care [5, 7, 9]. The fact that the MSK-HQ is a questionnaire for all MSK disorders might imply both weaknesses and strengths. Having one tool is a strength, because it helps to compare scores across different subgroups of MSK disorders. The location of the symptomatic body region might be of importance when using the MSK-HQ since people with different pain sites have different scores on the MSK-HQ [5]. The British study cohort consisted of 570 patients, including 150 hip patients, 150 knee patients, and 60 patients with shoulder problems. The mean total score of the MSK-HQ varied from 24.9 (hip), 27.5 (knee), to 33.5 (shoulder) [5]. Hence, it is important for clinicians to be aware of this variation among anatomical body regions when interpreting the MSK-HQ scores.

One potential weakness of our study is the choice of design with four weeks between our test and retest. A period of 1–2 weeks is suggested in the literature, unless a good reason for another time frame is presented [18]. One could argue that the mean (SD) of 31 (5.4) days between the tests was a too long period. Therefore, we included a global rating of change scale to determine a population for the retest that reported having “no change” in symptoms the last four weeks, which has shown acceptable in a previous study on the MSK-HQ [9]. Another potential limitation is that the participants volunteered to join the study which might influence the degree of generalisability to the whole population of people on sick leave. A strength of this study is the large sample which is substantially more than the lower limits of the recommendations for the different analyses [18].

## Conclusions

The results of this study suggest that the psychometric properties of the Norwegian version of the MSK-HQ in people on sick leave due to MSK disorders are good. The questionnaire seems appropriate for measuring domains related to MSK health in both research and clinical practice. Further investigations on different diagnoses and pain regions could be useful to determine differences in the total score.

## Data Availability

The datasets generated and analysed during the study will not be made publicly available due to national regulations.

## Appendix

### SPØRRESKJEMA OM MUSKEL- OG SKJELETTHELSE (MSK-HQ)

Dette spørreskjemaet handler om dine **ledd-, rygg-, nakke-, skjelett- og muskelsymptomer** slik som verking, smerter og/eller stivhet. Fokuser på den/de bestemte helseplagen(e) som du har søkt om behandling for her.

*For hvert spørsmål, ****kryss (✓) av én boks**** for å markere hvilket utsagn som*

*best beskriver din situasjon ****de siste to ukene****.*

**Table:**

1. Smerte/stivhet i løpet av dagen *(Pain/stiffness during the day)* Hvor intense har dine smerter og/eller stivhet i muskler/ledd vært på dagtid de siste to ukene? *(How severe was your usual joint or muscle pain and/or stifness overall during the day in the last 2 weeks)*			Ikke i det hele tatt *(Not at all)* _□4_	Litt *(Slightly)* _□ 3_	Moderat *(Moderat-ely)* _□ 2_	Ganske intense *(Fairly severe)* _□ 1_	Veldig intense *(Very severe)* _□ 0_
2. Smerter/stivhet om natten *(Pain/stiffness during the night)* Hvor intense har dine smerter og/eller stivhet i muskler/ledd vært om natten de siste to ukene? *(How severe was your usual joint or muscle pain and/or stiffness overall during the night in the last 2 weeks?)*			Ikke i det hele tatt *(Not at all)* _□ 4_	Litt *(Slightly)* _□ 3_	Moderat *(Moderat-ely)* _□ 2_	Ganske intense *(Fairly severe)* _□ 1_	Veldig intense *(Very severe)* _□ 0_
3. Gange *(Walking)* Hvor mye har symptomene hindret deg i å gå i løpet av de siste to ukene? *(How much have your symptoms interfered with your ability to walk in the last 2 weeks?)*			Ikke i det hele tatt *(Not at all)* _□ 4_	Litt *(Slightly)* _□ 3_	Moderat *(Moderat-ely)* _□ 2_	Veldig *(Severely)* _□ 1_	Har ikke kunnet gå *(Unable to walk)* _□ 0_
4. Vaske seg/påkledning *(Washing/Dressing)* Hvor mye har symptomene hindret deg i å vaske eller kle på deg de siste to ukene? *(How much have your symptoms* *interfered with your ability to wash or dress yourself in the last 2 weeks?)*			Ikke i det hele tatt *(Not at all)* _□ 4_	Litt *(Slightly)* _□ 3_	Moderat *(Moderat-ely)* _□ 2_	Veldig *(Severely)* _□ 1_	Klarer ikke å vaske eller kle på meg *(Unable to dress myself)* _□ 0_
5. Fysisk aktivitetsnivå *(Physical activity levels)* Hvor vanskelig har det vært for deg å være så fysisk aktiv som du ønsker (f.eks. å gå en tur eller jogge) pga. ledd/muskel-symptomene de siste to ukene? *(How much has it been a problem for you to do physical activities (e.g. going for a walk or jogging) to the level you want because of your joint or muscle symptoms in the last 2 weeks?)*			Ikke i det hele tatt *(Not at all)* □ 4	Litt *(Slightly)* □ 3	Moderat *(Moderat-ely)* □ 2	Veldig *(Very much)* □ 1	Ikke vært i stand til å være fysisk aktiv *(Unable* *to do physical activities)* □ 0
6. Arbeid/daglige aktiviteter *(Work/daily routine)* Hvor mye har dine ledd-/muskelsymptomer begrenset deg i ditt arbeid eller daglige aktiviteter de siste to ukene (inkludert jobb og husarbeid)? *(How much have your joint or muscle symptoms interfered with your work or daily routine in the last 2 weeks (including work & jobs around the house)?)*			Ikke i det hele tatt *(Not at all)* □ 4	Litt *(Slightly)* □ 3	Moderat *(Moderat-ely)* □ 2	Veldig *(Severely)* □ 1	Ekstremt *(Extremely)* □ 0
7. Sosiale aktiviteter og hobbyer *(Social activities and hobbies)* Hvor mye har dine ledd-/muskelsymptomer hindret deg i å være sosialt aktiv og å holde på med hobbyer de siste to ukene? *(How much have your joint or muscle symptoms interfered with your social activities and hobbies in the last 2 weeks?)*			Ikke i det hele tatt *(Not at all)* □ 4	Litt *(Slightly)* □ 3	Moderat *(Moderat-ely)* □ 2	Veldig *(Severely)* □ 1	Ekstremt *(Extremely)* □ 0
8. Behov for hjelp *(Needing help)* Hvor ofte har du trengt hjelp fra andre (inkludert familie, venner eller pleiere) på grunn av ledd-/muskelsymptomene de siste to ukene? *(How often have you needed help from others (including family, friends or carers) because of your joint or muscle symptoms in the last 2 weeks?)*			Aldri *(Not at all)* □ 4	Sjeldent *(Rarely)* □ 3	Noen ganger *(Someti-mes)* □ 2	Ofte *(Frequent-ly)* □ 1	Hele tiden *(All the time)* □ 0
9. Søvn *(Sleep)* Hvor ofte har du hatt problemer med søvn på grunn av ledd-/muskelsymptomene de siste to ukene? *(How often have you had trouble with either falling asleep or staying asleep because of your joint or muscle symptoms in the last 2 weeks?)*			Aldri *(Not at all)* □ 4	Sjeldent *(Rarely)* □ 3	Noen ganger *(Someti-mes)* □ 2	Ofte *(Frequent-ly)* □ 1	Hver natt *(Every night)* □ 0
10. Utmattelse eller lite energi *(Fatigue or low energy)* Hvor mye utmattelse eller mangel på energi har du følt de siste to ukene? *(How much fatigue or low energy have you felt in the last 2 weeks?)*			Ingen *(Not at all)* □ 4	Litt *(Slight)* □ 3	Moderat *(Modera-te)* □ 2	Veldig *(Severe)* □ 1	Ekstremt *(Extreme)* □ 0
11. Følelsesmessig velvære *(Emotional well-being)* I hvilken grad har du vært engstelig eller nedtrykt på grunn av dine ledd-/muskelsymptomer de siste to ukene? *(How much have you felt anxious or low in your mood because of your joint or muscle symptoms in the last 2 weeks?)*			Ingen *(Not at all)* □ 4	Litt *(Slightly)* □ 3	Moderat *(Moderat-ely)* □ 2	Veldig *(Severely)* □ 1	Ekstremt *(Extremely)* □ 0
12. Forståelse av dine helseplager og pågående behandling *(Understanding of your condition and any current treatment)* Når du tenker på ledd-/muskelsymptomene dine; hvor godt føler du at du forstår dine helseplager og din pågående behandling (inkludert din diagnose og medisinering)? *(Thinking about your joint or muscle symptoms, how well do you feel you understand your condition and any current treatment (including your diagnosis and medication)?)*			Fullstendig *(Complete-ly)* □ 4	Veldig godt *(Very well)* □ 3	Moderat *(Moderat-ely)* □ 2	Litt *(Slightly)* □ 1	Ikke i det hele tatt *(Not at all)* □ 0
13. Tro på å mestre symptomene dine *(Confidence in being able to manage your symptoms)* Hvor sikker har du følt deg i din egen mestring av ledd-/muskelplagene de siste to ukene (f.eks. medisinering eller endring av livsstil)? *(How confident have you felt in being able to manage your joint or muscle symptoms by yourself in the last 2 weeks (e.g. medication, changing lifestyle)?)*			Ekstremt *(Extremely)* □ 4	Veldig *(Very)* □ 3	Moderat *(Moderat-ely)* □ 2	Litt *(Slightly)* □ 1	Ikke i det hele tatt *(Not at all)* □ 0
14. Generell påvirkning *(Overall impact)* Samlet sett, hvor mye har dine ledd-/muskelsymptomer plaget deg de siste to ukene? *(How much have your joint or muscle symptoms bothered you overall in the last 2 weeks?)*			Ikke i det hele tatt *(Not at all)* □ 4	Litt *(Slightly)* □ 3	Moderat *(Moderat-ely)* □ 2	Veldig mye *(Very much)* □ 1	Ekstremt *(Extremely)* □ 0
Fysisk aktivitetsnivå _*(Physical activity levels)*_ Hvor mange dager i løpet av den siste uken har du vært fysisk aktiv i 30 minutter eller mer? Den fysiske aktiviteten må ha vært anstrengende nok til å øke pulsen din. Aktiviteten kan være idrett, trening, rask gange eller sykling. Husarbeid eller fysisk aktivitet som er en del av jobben din, skal ikke regnes med *(In the past week, on how many days have you done a total of 30 min or more of physical activity, which was enough to raise your heart rate? This may include sport, exercise and brisk walking or cycling for recreation or to get to and from places, but should not include housework* *or physical activity that is part of your job.)*							
Ingen dager *(None)* □	1 dag *(1 day)* □	2 dager *(2 days)* □	3 dager *(3 days)* □	4 dager *(4 days) □*	5 dager *(5 days) □*	6 dager *(6 days) □*	7 dager *(7 days) □*

## Abbreviations

CI: Confidence interval;
EQ-5D-5L: EuroQol 5 dimensions
HRQOL: Health-related quality of life
ICPC-2: International Classification of Primary Care, 2nd edition
ICC: Intraclass correlation coefficient
iPCQ: Institute for Medical Technology Assessment Productivity Cost Questionnaire
Keele STarT MSK: Keele Subgroups for Targeted Treatment tool
L01: Neck symptom/complaint
L02: Back symptom/complaint
L03: Low back symptom/complaint
L08: Shoulder symptom/complaint
L13: Hip symptom/complaint
L15: Knee symptom/complaint
L83: Neck syndrome
L84: Back syndrome without radiating pain
L85: Acuired deformity of spine
L86: Back syndrome with radiating pain
MSK-HQ: Musculoskeletal Health Questionnaire
NAV: Norwegian Welfare and Labor Administration
ÖMPSQ-SF: Örebro Musculoskeletal Pain Screening Questionnaire short form
PROMs: Patient-reported outcome measures
SEM: Standard error of measurement
SD: Standard deviation
SDC: Smallest detectable change
